# Assessment of Quality and Readability of Online Patient-Centered Arabic Web-Based Knowledge About Apicoectomy

**DOI:** 10.7759/cureus.48333

**Published:** 2023-11-05

**Authors:** Arwa Bafail, Rayan A Mohammad, Maher O Shahada, Anas M Alsaedi, Abdulmajid A Masoudi, Moataz E Karbouji, Muath S Alassaf

**Affiliations:** 1 Department of Restorative Dental Sciences, College of Dentistry, Taibah University, Madinah, SAU; 2 Department of Endodontics, Ohud Hospital, Madinah, SAU; 3 Dental Education, Taibah University, Madinah, SAU; 4 Dentistry, College of Dentistry, Taibah University, Madinah, SAU; 5 Department of Oral and Maxillofacial Surgery, King Fahad General Hospital, Madinah, SAU; 6 Orthodontics and Dentofacial Orthopedics, Taibah University, Madinah, SAU

**Keywords:** readability, fkgl, discern tool, endodontic micro-surgery, endodontic surgery, apical surgery, apicoectomy

## Abstract

Background

Endodontic microsurgery (apicectomy) can be considered in cases of persistent infection that is resistant to conventional root canal treatment. The aim of this study was to evaluate the quality and readability of the available online information regarding the apicectomy procedure in Arabic.

Methods

Online search on the three most commonly used websites (Google, Yahoo, and Bing) using one keyword. The first 100 websites from each search were analyzed for quality and readability using DISCERN instrument scores, the Journal of the American Medical Association (JAMA) benchmarks, the Health On the Net (HON) seal, Flesch Reading Ease Scores (FRES), Flesch-Kincaid Grade Level (FKGL), and the Simplified Measure of Gobbledygook (SMOG) Index.

Results

Searching using the Arabic translation for "root end resection surgery" revealed 349,900 websites. Following the inclusion criteria, 31 websites were selected and evaluated in this study. The selected websites belonged to either non-profit organizations or commercial websites. The quality of most of the selected websites received a moderate score (83.9%) using the DISCERN tool. None of the selected websites obtained the HON seal. Quality evaluation using the JAMA benchmarks revealed that currency was the most achieved item (45.2%), followed by authorship (22.6%). Evaluation of the readability of the selected websites using the FRES, FKGL, and SMOG showed that the included websites were considered readable.

Conclusion

Although the included websites were readable, the quality of the websites was moderate. There is an urgent need to create more trustworthy and readable websites explaining the different endodontic treatments.

## Introduction

Root canal treatment (endodontic treatment) is a dental procedure that encompasses a number of treatments that are concerned with preventing and treating inflamed and infected pulp tissues that must be removed from the root canal system to prevent further problems [1]. There is one specific set of aims for endodontic treatment, which is to prevent or cure periradicular periodontitis [1]. This is accomplished through multiple stages of root canal treatment, including diagnosis, treatment planning, access cavity, instrumentation with mechanical and chemical aids, and complete obturation with a filling material, with a reported success rate of 92.6% [2]. Complications may arise at any stage of the primary endodontic treatment that can affect the treatment outcome [3]. When the infected areas cannot be identified, it becomes the major cause of disease persistence and endodontic failure [4]. In the presence of endodontic failure, secondary root canal treatment (root canal retreatment) is required, with a reported success rate of 77% [5].

Surgical endodontic treatment (apicoectomy) is performed to save the natural teeth when conservative endodontic treatment has failed [6]. In this surgery, the tip of the root or roots is surgically removed using biocompatible materials for retrograde closure, as the primary goal of the surgery is to prevent bacterial microleakage from the root canal system into the periradicular tissues [7]. The overall survival rate for apicoectomy was 88% after 72 months [8]. Different techniques can be used for apicoectomy with different retrograde filling materials; modern apicoectomy has a five-fold higher success rate than traditional apicoectomy [9]. The apicoectomy outcome can be affected by the technique used, as clinicians advise using magnification with microsurgical techniques, i.e., conservative incision design and gentle flap elevation, preparation of a small osteotomy, and using sonic or ultrasonic-driven microtips, all of which can result in faster postsurgical healing since less trauma is caused to the patient [10]. Also, the accuracy of the apicoectomy procedure can be increased by using a digitally planned 3D-printed guide by taking an intraoral scan of the patient with a 3D image of the patient using cone beam computed tomography (CBCT) [11]. The ideal root end-filling material has yet to be found, even though there are at least 19 different materials available [12]. The most frequently used material in the apicoectomy procedure was amalgam restoration, with a satisfying result in many cases [12]. More suitable materials give better results than amalgam restoration in apicoectomy procedures, such as mineral trioxide aggregate (MTA), intermediate restorative materials (IRM), and super-ethoxy benzoic acid [12].

Nowadays, online information has become a highly reliable source for finding answers to all people’s questions [13]. People seek health information online for a variety of reasons, including reassurance, to find alternative opinions regarding medical interventions, and to better understand the information provided in a clinical setting [14]. There remains a concern regarding the quality, accuracy, and readability of online health-related information, despite its abundance and availability [15]. As there is only one study that evaluated the quality of online information about apicoectomy on English websites, there has not been a study evaluating online information about apicoectomy on Arabic websites [16]. Thus, this study seeks to bridge the knowledge gap regarding the apicoectomy procedure on Arabic websites and evaluate the quality and readability of the information available.

## Materials and methods

Searching strategy

Searching with the Arabic translation for “root end resection surgery” as "استئصال قمة جذر السن" was carried out on the 24th of August 2023 using Bing, Google, and Yahoo search engines. This study was conducted at Taibah University at the College of Dentistry, Madinah, Saudi Arabia. The first 100 websites that appeared through Bing, Google, and Yahoo search engines for the term were explored to evaluate the content, quality, and readability of the available information. Even though most users only look at the first page of Google, we included additional websites beyond the first page [16]. The list of websites was thoroughly screened for any non-working links or duplicates. The exclusion criteria applied involved scientific articles, pure video, pure audio, websites with no content related to apicoectomy, textbooks, social media/forums, websites that denied direct access to the content, non-Arabic websites, and advertisements. The remaining websites were categorized according to Ní Ríordáin and McCreary (2009) based on affiliation (non-profit organization, commercial, university/medical center, and government), specialization (partly related, exclusively related), content type (question and answer, human interest stories, medical facts, clinical trials), and content presentation (image, video, and audio).

Quality assessment

The quality of the online information was assessed using the DISCERN instrument [17], the Journal of the American Medical Association (JAMA) benchmarks for website analysis [18], and Health On the Net (HON) [12]. DISCERN is a validated 16-point questionnaire developed by the University of Oxford to examine the reliability (questions 1 to 8) and specific details of information on treatment choices (questions 9 to 15), plus an additional question for the overall quality rating (question 16) [17]. Each question is rated on a numerical scale from 1 to 5 (1 = very poor, 2 = poor, 3 = moderate, 4 = good, 5 = excellent).

JAMA benchmarks were used to analyze the quality of the website. These benchmarks require clarity about content authorship, including authors and contributors, their affiliations, and relevant credentials; an attribution list of references and sources of information; disclosure of website ownership, sponsorship, advertising, commercial funding arrangements, conflicts of interest, and currency, i.e., dates of content posted and updated [18].

HON is a non-profit organization founded in 1995 that promotes reliable and transparent health information online. It gives accreditation to websites that follow the HON code of ethical conduct, consisting of eight principles, including authority, privacy, attribution, transparency, financial disclosure, and advertising policy [18].

Readability assessment

The readability was assessed using four different measures: Flesh Reading Ease Scores (FRES), Flesh-Kincaid Grade Level (FKGL), and the Simplified Measure of Gobbledygook (SMOG). The Flesch Reading Ease (FRES) formula, developed in the 1940s, measures the readability of text by considering the average sentence length and the average number of syllables per word. A higher score indicates that the text is easier to read. A score of more than 90 equates to the reading age of a ten-year-old, while a score of 30-49 equates to the reading age of an adult. The FKGL is based on the average number of words per syllable and sentence, while the SMOG Index takes into account the number of polysyllabic words per sentence [19]. The text will be considered readable for FKGL and SMOG if it equals seven or less. Regarding FRES, the acceptable score is set at 80 or more.

Data analysis plan

Data were collected and recorded in Microsoft Office Excel (Microsoft Corporation, Redmond, Washington, United States) spreadsheets. Descriptive statistical analyses were calculated using IBM SPSS Statistics for Windows, Version 26.0 (released 2019; IBM Corp., Armonk, New York, United States). Data are presented as mean with standard deviation for quantitative data and as frequency and percentages for categorical data.

Ethical considerations

In conducting this study, we have exclusively employed publicly available data that does not include personally identifiable information, confidential data, or other sensitive content related to individual patients or participants. As such, the nature of the study design doesn’t require obtaining informed consent or undergoing formal ethical review processes generally mandated for research involving human or animal subjects.

## Results

Available websites

**Figure 1:**
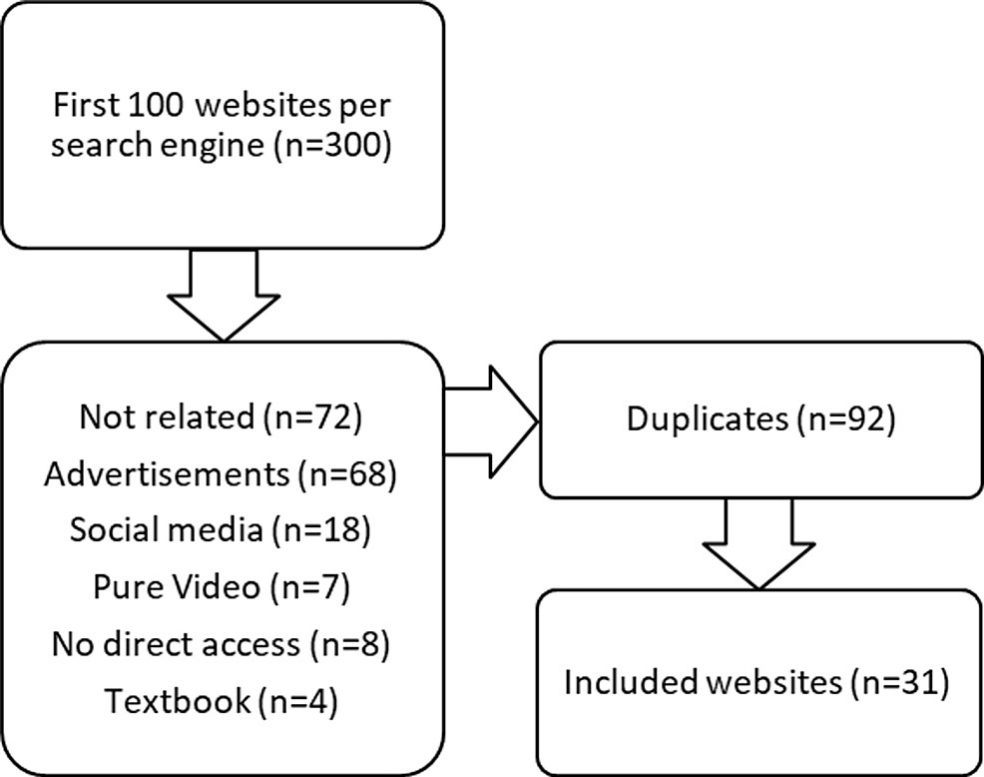
Flow chart of the search strategy

When searching for the Arabic term for "root end resection surgery" on search engines, Google found 218,000 websites, Bing found 48,900, and Yahoo found 83,000. We evaluated the first 100 results from each engine for eligibility criteria and found 31 websites that fit the criteria. The remaining 269 websites were excluded. See Figure 1 for the selection strategy.

Checked websites were categorized, and it was found that most of them (21, or 67.7%) belonged to non-profit organizations, followed by commercial websites (10, or 32.3%). None of the websites were affiliated with the government, universities, or medical centers. All 31 websites contained medical content, with two (6.5%) including Q&A and only one (3.2%) including clinical trials. Five (16.1%) websites included human interest stories. Seventeen (54.8%) websites included images, six (19.4%) included videos, and only two included audio (6.5%). For a summary of the website's affiliation, content type, and presentation, see Table 1.

**Table 1:** Categorization of websites based on affiliation, specialization, content type and content presentation (n=31)

Category	Criteria	Number of websites	Percentage
Affiliation	Commercial	10	32.3
	Non-profit organization	21	67.7
	University/medical center	0	0
	Governmental	0	0
Specialization	Exclusively related	0	0
	Partly related	31	100
Content type	Medical facts	31	100
	Clinical trials	1	3.2
	Human interest stories	5	16.1
	Question and answer	2	6.5
Content presentation	Image	17	54.8
	Video	6	19.4
	Audio	2	6.5

Quality assessment

Out of the 31 websites examined, the average DISCERN score for all of them was 2.90 (± 0.58). However, no website achieved the maximum score of 80 or the minimum score of 16. The poorest scores were for the twelfth question, "Does it describe what would happen if no treatment is used?" with only one website achieving a score of 25 (80.6%), and the fourth question, "Is it clear what sources of information were used to compile the publication?" with only 21 websites achieving a score of 67.7%. Most of the websites received moderate scores, with 26 (83.9%) falling into this category, while only five (16.1%) received low scores, and none achieved high scores. Table 2 summarizes the DISCERN analysis for each question, and no website obtained the HON seal.

**Table 2:** Means and standard deviation scores for DISCERN instrument (n=31) Data presented as mean with standard deviation (SD), maximum (Max), and minimum (Min)

Domain	DISCERN question	Mean (SD)	Max	Min
Reliability	Q1. Explicit aims	3.35 (1.26)	5	1.5
	Q2. Aims achieved	3.35 (1.24)	5	1.5
	Q3. Relevance	4.48 (0.81)	5	1.5
	Q4. Explicit sources	1.71 (1.51)	5	1
	Q5. Explicit date	1.82 (1.29)	5	1
	Q6. Balanced and unbiased	2.65 (0.64)	4.5	1.5
	Q7. Additional sources	1.94 (1.10)	4	1
	Q8. Areas of uncertainty	2.16 (0.44)	3	1.5
Treatment options	Q9. How treatment works	3.65 (1.13)	5	1
	Q10. Benefits of treatment	2.69 (0.95)	4.5	1
	Q11. Risk of treatment	2.92 (1.19)	4.5	1
	Q12. Effects of no treatment	1.47 (0.60)	3	1
	Q13. Effects on quality of life	2.06 (0.68)	3.5	1
	Q14. All alternatives described	1.45 (0.72)	3.5	1
	Q15. Shared decision	1.69 (1.08)	4.5	1
Overall rating		2.90 (0.58)	4	2

When interpreting JAMA benchmarks, currency was the most achieved item in 14 (45.2%) websites, followed by authorship in seven (22.6%) websites, and attributions in six (19.4%) websites. Disclosure was the least achieved item, with only three (9.7%) websites meeting this requirement. No website received a full score in the JAMA benchmarks. Table 3 shows the number of achieved JAMA items per website. Table 4 displays the distribution of DISCERN and JAMA scores among the included websites.

**Table 3:** Summary of the quality and readability assessment of the included websites (n=31) JAMA: Journal of the American Medical Association; HON: the Health On the Net

Item	Frequency	%
JAMA Benchmarks		
Authorship	7	22.6
Attribution	6	19.4
Disclosure	3	9.7
Currency	14	45.2
JAMA per website		
No item achieved	11	35.5
One item achieved	11	35.5
Two items achieved	8	25.8
Three items achieved	1	3.2
Four items achieved	0	0.0
HON Code		
Obtained	0	0.0
Not Obtained	31	100
DISCERN Score		
Low	5	16.1
Moderate	26	83.9
High	0	0
Flesch Reading Ease		
≥ 80	30	96.8
< 80	1	3.2
Flesch Kincaid Grade Level		
≤7	27	87.1
>7	4	12.9
Simple Measure of Gobbledygook		
≤7	31	100
>7	0	0

**Table 4:** Distribution of JAMA and DISCERN categories based on website affiliation (n=31) Data presented as frequency and percentage (%) JAMA: Journal of the American Medical Association

Variable	Commercial	Non-profit organization	Total
JAMA items			
None	2 (6.5%)	9 (295)	11 (35.5%)
One	5 (16.1%)	6 (19.3%)	11 (35.5%)
Two	3 (9.7%)	5 (16.1%)	8 (25.8%)
Three	0	1 (3.2%)	1 (3.2%)
DISCERN			
Low	1 (3.2%)	4 (12.9%)	5 (16.2%)
Moderate	9 (29%)	17 (54.8%)	26 (83.9%)
High	0	0	0

Readability assessment

According to Table 3, the FRE scale rating for the websites analyzed ranged from 79.7 to 100, with an average of 99.2 (± 6.9). The FKGL varied from 0.2 to 12.6, with a total average of 4.2 (± 2.7). The SMOG rating had a range of 1.8 to 2.9, with an average of 2.1 (± 0.4). A total of 30 (96.8%) websites scored 80 or above on the FRE scale, and 27 (87.1%) websites scored seven or less on the FKGL. All websites scored 7 or less on the SMOG rating.

## Discussion

The aim of endodontic treatment is to cure or prevent apical periodontitis [20]. Epidemiological studies reported that about one-third to two-thirds of root canal-treated teeth have persistent periapical lesions that necessitate further intervention [21]. Non-surgical root canal retreatment should be the first treatment option. However, endodontic microsurgery (apicectomy) could be considered in some cases, such as persistent lesions not responding to conventional root canal treatment, apical cysts, extrardicular infections, the presence of irretrievable posts, or in cases of complex anatomy [22]. With the increased use of technology, patients are increasingly seeking advice and information from online resources. It is imperative to have accurate and simple information to prevent misleading the patient with inaccurate information. This study, to the best of our knowledge, is the first to evaluate the quality of the available information on the internet regarding the apicectomy procedure published in Arabic.

In this study, the most commonly used search engines, including Google, Yahoo, and Bing, were used. A search using the Arabic translation for "root end resection surgery" revealed 349,900 websites from the three search engines. The first 100 websites from each search engine were carefully evaluated using the eligibility criteria. Following the elimination of duplicates, 31 websites were selected, with the main reasons for exclusion being that they were not related to the topic or were advertisements. Most of the included websites belong to non-profit organizations, followed by commercial websites. All the included websites were partly related to the topic of presenting medical facts about the procedure using pictures or videos. It is difficult for the layperson to understand the concept of the apicectomy procedure. Thus, the presence of trustworthy, readable websites is crucial to simplifying the information for patients. This study highlights the need for more detailed and trustworthy websites.

DISCERN is one of the most commonly used tools for the assessment of the quality of medical websites [23]. Assessment of the quality of the included websites using the DISCERN tool revealed that most websites are of moderate quality (83.9%), with the poorest scores related to questions about treatment options (question 12 and 14). In studies evaluating oral maxillofacial pathology, the quality of web-based information regarding oral lesions was poor [24]. Other studies evaluating the web-based dental implant information reported poor overall quality [23]. In a previous study evaluating online information about apicectomy, the quality of the included websites was not evaluated [16]. Using the JAMA tool, none of the included websites fulfilled the four JAMA benchmark items. About one-third of the included websites did not achieve any of the four JAMA benchmark items. The currency was the most achieved item, while disclosure was the least achieved item.

None of the included websites obtained the HON seal. The HONcode certification may be absent on health websites for various reasons. Financial constraints often pose a significant barrier, with some websites unable to afford the associated fees. Compliance with HONcode principles demands ongoing effort, potentially overwhelming smaller or non-profit sites. Navigating the complexities of data privacy and content standards can be challenging, necessitating technical expertise. Moreover, HONcode guidelines may evolve over time, necessitating continuous adaptation. Autonomy is another factor; some websites prefer not to adhere to external standards, choosing to maintain control over their content and practices. Additionally, limited awareness about HONcode certification exists, leading some websites to overlook its potential benefits.

Information targeting the general population should be presented in simple and clear terms so a layperson can grasp the information. Common readability tests use the average sentence length, the number of words, and the number of syllables per word to calculate the readability of a given text. Standard, well-established readability tests include FRES, FKGL, and SMOG. The text is considered readable in FKGL and SMOG if it scores seven or less, while for FRES it should score 80 or more [19]. Based on the tools used, the included websites were considered readable. Previous studies evaluating the readability of dental health information on various topics revealed that most of the included websites were difficult to read, targeting healthcare professionals and urging the need for simple information that the majority of patients can understand [16,23,25,26]. In a study evaluating the readability of online information about apicectomy in the English language, they reported a high level of difficulty [16]. This contradicts the results of the current study, which found that most of the included websites were easy to read [27,28]. This might be due to the difference in language, as most of the medical terminology used is in English. In the Arabic language, the terminology used is understandable to the general population.

Limitations of the study 

In relation to our study's limitations, using one search term may limit the generalizability of the study findings, although we have included the first hundred results per search engine. We also focused on website content and excluded social media and videos. However, most of the tools and indices available are mainly intended to measure the quality and readability of written material. In addition, this study evaluated the Arabic content only; while this is the aim of the study, including two languages may improve the findings in future studies.

## Conclusions

This study aimed to evaluate the quality and readability of available web-based information about apicectomy published in Arabic. Although the included websites were readable, the quality of the websites was moderate. There is an urgent need to create more trustworthy and readable websites explaining the different endodontic treatments.
